# The effect of intravesical Bacillus Calmette-Guerin (BCG) treatment on sperm parameters in non-muscle-invasive bladder cancer patients

**DOI:** 10.1186/s12610-025-00259-0

**Published:** 2025-05-15

**Authors:** Sergen Sahin, Ismail Ulus, Ibrahim Ogulcan Canitez, Serhat Yentur, Mustafa Zafer Temiz, Atilla Semercioz

**Affiliations:** Bagcilar Research and Training Hospital, Urology Clinic, Istanbul, Türkiye

**Keywords:** Bacillus Calmette, Guerin, Non, Muscle, Invasive bladder cancer, Sperm parameters, Semen quality, Fertility, Intravesical therapy, Bacille de Calmette-Guérin, Cancer de Vessie non invasif sur le plan musculaire, Paramètres du Sperme, Qualité du Sperme, Fertilité, Thérapie intra vésicale

## Abstract

**Background:**

Bladder cancer is the sixth most common cancer in males. Bacillus Calmette-Guerin (BCG) immunotherapy, the standard for non-muscle-invasive bladder cancer (NMIBC), effectively reduces recurrence and progression. This study examines the impact of intravesical BCG therapy on semen parameters, focusing on hormonal profiles and sperm quality.

**Results:**

This prospective study included 23 sexually active males diagnosed with NMIBC receiving intravesical BCG therapy. Semen analyses were performed prior to treatment and three months following the induction course. Hormonal profiles, comprising total testosterone (TT), follicle-stimulating hormone (FSH), luteinizing hormone (LH), and prolactin (PRL), were evaluated. Patients underwent six weekly instillations of 80 mg/ml SII-ONCO-BCG®, commencing four weeks post-TUR-B surgery. Data from clinical, radiological, and laboratory sources were gathered for analysis. Post-treatment evaluation indicated a significant reduction in sperm concentration, total sperm count, progressive motility, and normal morphology (*p* < 0.05), alongside a notable increase in the percentage of immotile sperm. LH and PRL levels remained stable despite these changes, while FSH levels exhibited a significant increase (*p* < 0.05).

**Conclusion:**

BCG therapy adversely affects sperm quality, leading to a marked decrease in sperm concentration, total sperm count, progressive motility, and normal morphology. The findings underscore the potential gonadotoxic effects of BCG, necessitating fertility counseling prior to treatment initiation, especially in younger patients. Semen cryopreservation should be regarded as a preventive strategy. While BCG is considered the gold standard for NMIBC treatment, additional long-term studies are necessary to evaluate the reversibility of its effects and to investigate alternative intravesical therapies that present lower reproductive risks.

## Résumé

**Contexte:**

Le cancer de la vessie est le sixième cancer le plus fréquent chez les hommes. L’immunothérapie par bacille de Calmette-Guérin (BCG), la norme pour le cancer de la vessie non invasif sur le plan musculaire (NMIBC), en réduit efficacement la récidive et la progression. Cette étude examine l’impact du traitement intra vésical par BCG sur les paramètres du sperme, en se concentrant sur les profils hormonaux et la qualité du sperme.

**Résultats:**

Cette étude prospective a porté sur 23 hommes sexuellement actifs diagnostiqués avec un NMIBC recevant un traitement BCG par voie intra vésicale. Des analyses de sperme ont été effectuées avant le traitement et trois mois après le cycle d’induction. Les profils hormonaux, incluant la testostérone totale (TT), l’hormone folliculo-stimulante (FSH), l’hormone lutéinisante (LH) et la prolactine (PRL), ont été évalués. Les patients ont reçu six instillations hebdomadaires de SII-ONCO-BCG® à 80 mg/ml, à partir de quatre semaines après la chirurgie TUR-B. Les données provenant de sources cliniques, radiologiques et de laboratoire ont été recueillies aux fins d’analyses. L’évaluation post-traitement a révélé une réduction significative de la concentration de spermatozoïdes, du nombre total de spermatozoïdes, de la motilité progressive et de la morphologie normale (*p* <0,05), ainsi qu’une augmentation notable du pourcentage de spermatozoïdes immobiles. Les taux de LH et de PRL sont restés stables malgré ces changements, tandis que les taux de FSH ont montré une augmentation significative (*p* <0,05).

**Conclusions:**

Le traitement par le BCG affecte négativement la qualité du sperme, entraînant une diminution marquée de la concentration de spermatozoïdes, du nombre total de spermatozoïdes, de la motilité progressive et d’une morphologie normale. Les résultats soulignent les effets gonadotoxiques potentiels du BCG, nécessitant des conseils en matière de fertilité avant le début du traitement, en particulier chez les patients plus jeunes. La cryoconservation du sperme doit être considérée comme une stratégie préventive. Bien que le BCG soit considéré comme l’étalon-or pour le traitement des NMIBC, des études supplémentaires à long terme sont nécessaires pour évaluer la réversibilité de ses effets et pour étudier d’autres thérapies intra vésicales qui présentent des risques reproductifs plus faibles.

## Background

Bladder cancer is the sixth most prevalent cancer in males and ranks as the ninth most common cancer when both genders are considered. It stands as the second most common malignancy of the urogenital system, following prostate cancer [1]. While bladder cancer is generally more frequent in older individuals, its incidence is increasing among young and middle-aged adults [2].

Bacillus Calmette-Guerin (BCG) immunotherapy is the gold standard treatment modality in reducing recurrence and progression rates in non-muscle-invasive bladder cancer (NMIBC) [3]. BCG induces an anti-tumoral effect by generating a T cell-mediated immune response in the bladder. Adverse effects related to intravesical BCG instillation can range from common, mild, and transient symptoms (such as dysuria and flu-like symptoms) to more severe and rarely occurring complications, which can be life-threatening [4]. These adverse effects are thought to result from both local and systemic immunomodulatory mechanisms, which can impact various organs and physiological systems [5].

Despite the well-established efficacy of BCG in bladder cancer treatment, its potential impact on male fertility remains underexplored. Most previous studies have been limited by small sample sizes, a narrow focus on sperm concentration, or insufficient analysis of sperm morphology and motility. In contrast, our study provides a comprehensive evaluation of sperm parameters, including morphology and motility, while also investigating the potential immunological mechanisms underlying these effects.

Additionally, male infertility is influenced by various factors, including smoking, alcohol consumption, obesity, genetic predispositions, hormonal imbalances, and environmental pollutants [6]. Considering these variables enhances the reliability of our findings and allows a more holistic interpretation of the observed changes.

The aim of this study is to assess the impact of intravesical BCG therapy on semen parameters in men diagnosed with non-muscle-invasive bladder cancer, offering valuable insights into its potential reproductive consequences.

## Materials and methods

The research was conducted ethically in accordance with the World Medical Association Declaration of Helsinki. A prospective single-center study was performed, with Institutional Review Board approval granted under the ID 2022/11/11/343. Clinical notes, laboratory, and pathology data were collected from our institution.

We enrolled sexually active male patients diagnosed with NMIBC and indicated for intravesical BCG therapy from July 2022 to January 2023, based on their sequential presentation. We excluded patients with diabetes mellitus, genital surgery, specific medications affecting spermatogenesis, chemotherapy, and radiotherapy histories. Patients were informed orally about the study and provided written consent. Treatment began 4 weeks after TUR-B surgery and involved a 6-week induction course of intravesical BCG immunotherapy. Each treatment session involved the use of 80 mg/ml of SII-ONCO-BCG® (Serum Institute of India PVT. LTD., India) preparation.

A post-hoc power analysis was performed to assess the adequacy of our sample size (n = 23) for detecting significant differences in sperm parameters. The analysis was conducted with a statistical power of 80% and a significance level of 5% (α = 0.05). The minimum required sample size was determined to be 8 subjects based on these parameters. The study exhibited adequate power to identify statistically significant alterations in sperm concentration and motility, indicating that the sample size, although relatively small, was sufficient to detect clinically relevant effects.

Semen analysis was performed according to the WHO 6th Edition (2021) guidelines [7]. Sperm morphology was assessed using Kruger’s strict criteria, a well-established method for evaluating morphological abnormalities.

We measured total testosterone (TT), prolactin (PRL), luteinizing hormone (LH), and follicle-stimulating hormone (FSH) levels one week before the first dose of BCG induction therapy and three months after the last dose. Blood samples were collected between 8:30 AM and 10:30 AM after an 8-h fast. The samples were centrifuged at 4000 rpm for 10 min to separate the serum, which was then analyzed for parameter levels using the UniCel™ DxI 800 Access Immunoassay System (Beckman Coulter Inc., Brea, CA, USA) in the same laboratory each time.

A semen analysis was performed on each patient one week before the initiation of intravesical BCG instillations. We conducted a repeat semen analysis three months after the BCG induction therapy to assess the potential impact of intravesical BCG immunotherapy on semen parameters. Semen analyses were conducted in the same laboratory. We collected the samples after 4 days of sexual abstinence. Patients provided semen samples via masturbation in a special room equipped with sterile plastic containers in the andrology laboratory. We incubated the semen at 37°C until it liquefied.

Macroscopic evaluation assessed pH, liquefaction, viscosity, appearance, and volume. We measured the pH with a disposable pH paper and the volume with a graduated pipette. In the first microscopic evaluation, mucus thread formation, sperm aggregation or agglutination, the presence of non-sperm cells, motility, and sperm count were assessed. We thoroughly mixed the samples to prevent sedimentation, and systematically examined the prepared wet mounts under a binocular microscope (Olympus CX31) at 200 × or 400 × magnification.

The total spermatozoa count was calculated based on the concentration measured during semen evaluation using an improved Neubauer hemocytometer. For sperm morphology evaluation, the semen smear was air-dried, fixed, and stained with Diff-Quick. We examined the morphology under an oil immersion lens at 1000 × magnification, and calculated the percentage of normal and abnormal forms for 200 spermatozoa.

### Statistical analysis

We used descriptive statistics like mean, standard deviation, median, minimum, maximum, frequency, and ratio values in the data analysis. The distribution of variables was assessed using the Kolmogorov–Smirnov test. We employed paired sample t-tests and Wilcoxon tests to analyze the dependent quantitative data. Spearman correlation analysis was utilized for correlation analysis. A p-value < 0.05 was considered statistically significant. SPSS (Statistical Package for the Social Sciences) version 28.0 (IBM SPSS Inc., Chicago, IL) was used for data analysis.

## Results

The study were included 23 patients, with a mean age of 54.7 ± 6.3 years (range: 35–61). Among them, Ta high-grade (HG) was the most prevalent tumor type, followed by Ta low-grade (LG), T1 HG, T1 HG + carcinoma in situ (CIS), and primary CIS (Table 1). Hypertension, chronic obstructive pulmonary disease, and ankylosing spondylitis were among the conditions that the remaining patients had, while over half of the patients (56.5%) had no comorbidities (Table 1).

**Table 1:** Distribution of patient and tumor characteristics in the study cohort

Age (years) (Mean ± SD, Min–Max)	54.7 ± 6.3	35–61
Stage at TUR-B	n	%
CIS	1	4.3%
T1 HG	4	17.4%
T1 HG + CIS	6	26.1%
Ta HG	9	39.1%
Ta LG	3	13.0%
Concomitant disease		
None	13	56.5%
AS	1	4.35%
HT	6	26.1%
COPD	3	13.05%

This table presents the demographic and clinical characteristics of the study participants, including tumor stage at transurethral resection of the bladder tumor (TUR-B) and the presence of comorbidities. Data are expressed as median (min–max) for continuous variables and as frequencies (%) for categorical variables

*CIS* Carcinoma in situ, *HG* High grade, *LG* Low grade, *TUR-B* Transurethral resection of bladder tumor, *AS* Ankylosing Spondylitis, *HT* Hypertension, *COPD* Chronic obstructive pulmonary disease

All patients completed the 6-week course of intravesical BCG instillations. During the treatment, seven patients experienced minor complaints such as increased urinary frequency and dysuria, which were managed conservatively.

Post-treatment, TT and FSH levels exhibited a significant increase compared to pre-treatment values (*p* < 0.05). No significant changes were observed in LH and PRL levels (*p* > 0.05). TT and FSH levels exhibited a consistent upward trend, whereas LH and PRL levels remained relatively stable during the study period (Table 2). The findings indicate that intravesical BCG treatment may significantly influence TT and FSH levels, while LH and PRL levels seem to remain unchanged (Table 2).

**Table 2:** Comparison of hormonal levels before and after intravesical BCG instillation

Variables	Pre-BCG	Post-BCG	*p* value
Total Testosterone (ng/mL, Mean ± SD)	3.8 ± 1.5	4.5 ± 1.9	0.029**
FSH (IU/L, Median ± IQR)	8.7 ± 6.4	9.5 ± 7	0.037*
LH (IU/L, Median ± IQR)	10.2 ± 13.2	10.2 ± 13.3	0.808*
Prolactin (mcg/L, Mean ± SD)	11.6 ± 4.5	12 ± 3.9	0.729**

This table compares pre- and post-treatment hormonal levels in patients undergoing intravesical BCG therapy. Wilcoxon test was used for non-normally distributed variables, and paired t-test was used for normally distributed variables. Data are expressed as mean ± standard deviation (SD) for normally distributed variables and as median ± interquartile range (IQR) for non-normally distributed variables

*BCG* Bacillus Calmette-Guerin, *FSH* Follicle-stimulating hormone, *LH* Luteinizing hormone, *SD* Standard deviation, *IQR* Interquartile range

^*^Wilcoxon test was applied

^**^Paired sample t-test was applied

Intravesical BCG therapy resulted in a substantial decrease in sperm concentration, total sperm count, and progressive motility (*p* < 0.05). Furthermore, the proportion of immotile sperm experienced a substantial increase following the treatment. Four patients developed de novo oligospermia (< 16 million spermatozoa/ml), which is particularly noteworthy. Post-treatment, there was also a significant decline in the morphology of normal sperm (*p* < 0.05) (Table 3).

**Table 3:** Comparison of semen parameters before and after intravesical BCG treatment

Semen paremeters	Pre-BCG	Post-BCG	*p* value
Semen volume (mL, Median ± IQR)	2.0 ± 1.1	1.9 ± 0.8	0.748*
pH (Median ± IQR)	8.0 ± 0	8.0 ± 0	1.000*
Sperm concentration (10^6^/mL, Mean ± SD)	67.0 ± 40.8	44.9 ± 31.7	0.001**
Total sperm count (10^6^ per ejaculate, Median ± IQR)	124.3 ± 75.2	84.3 ± 68.8	0.016*
Total motility (%, Median ± IQR)	52.5 ± 38.3	23.1 ± 27.1	0.001*
Progressive motility (%, Mean ± SD**)**	38.0 ± 16.1	24.7 ± 16.4	0.001**
Non progressive motility (%, Median ± IQR)	9.2 ± 2.1	9.1 ± 2.8	0.979*
Immotile sperm (%, Mean ± SD)	52.8 ± 16.5	66.2 ± 17.2	0.001**
Leukocytes (10^6^/mL, Median ± IQR)	0.26 ± 0.45	0.17 ± 0.39	0.480*
Normal morphology (%, Median ± IQR)	1.57 ± 1.04	0.26 ± 0.54	0.001*

This table presents the changes in semen parameters before and after BCG therapy, assessing volume, motility, sperm concentration, and morphology. Wilcoxon test was used for non-normally distributed variables, and paired t-test was used for normally distributed variables. Values are expressed as median ± interquartile range (IQR) for non-normally distributed variables and as mean ± standard deviation (SD) for normally distributed variables

*BCG* Bacillus Calmette-Guerin, *SD* Standard deviation, *IQR* Interquartile range

^*^Wilcoxon test was applied

^**^Paired sample t-test was applied

Nevertheless, no statistically significant correlation was observed between patient age and pre- and post-treatment variations in sperm morphology, sperm concentration, total sperm count, total progressive motile sperm count, or semen volume (*p* > 0.05) (Table 3).

**Fig. 1:**
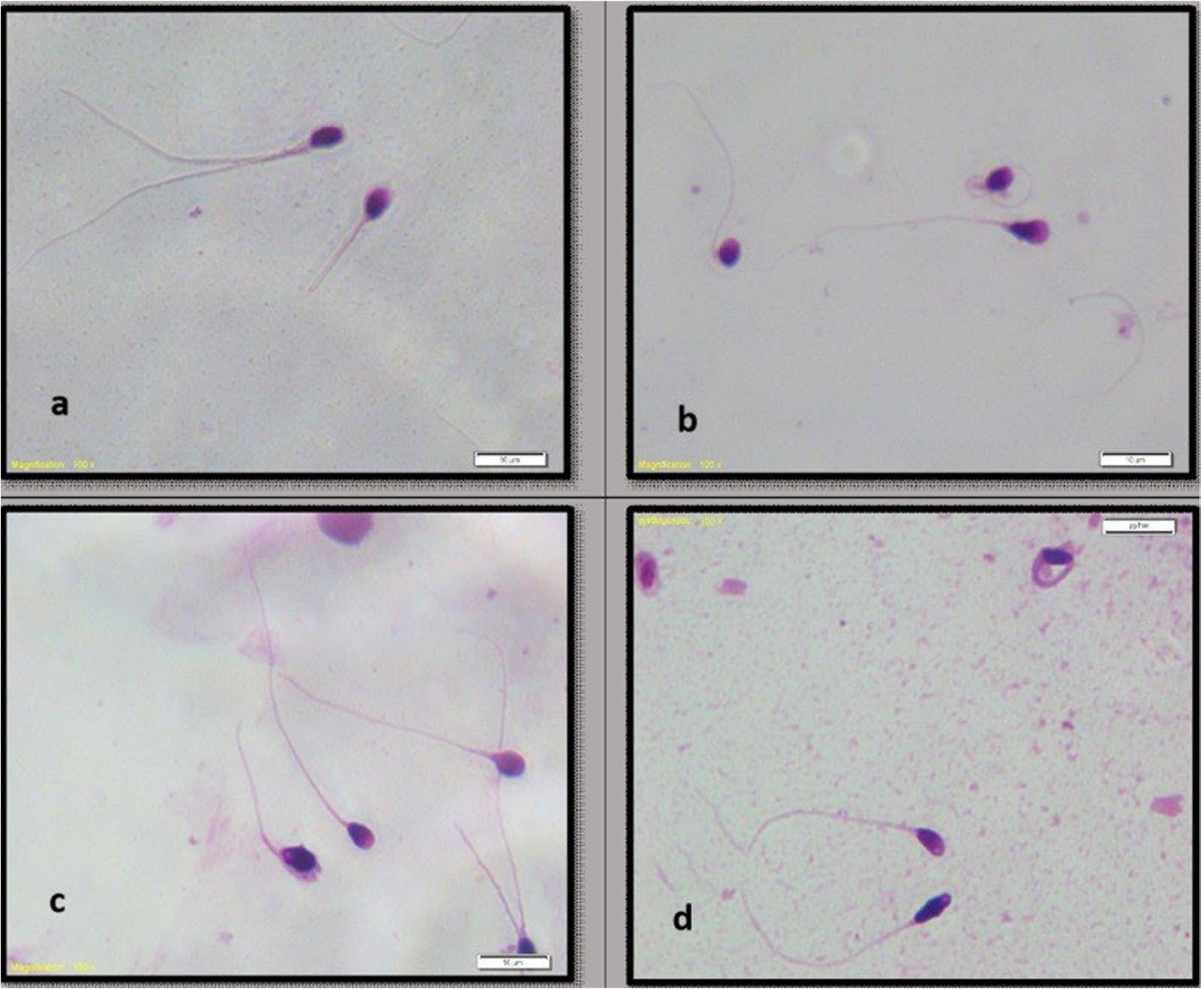
Morphological abnormalities in sperm cells after intravesical BCG therapy. Representative Papanicolaou-stained images of sperm morphological defects observed after intravesical Bacillus Calmette-Guérin (BCG) therapy. **a**-**c** Short tail defect, **b** Bent neck defect, **d** Amorphous head defect. These abnormalities were identified using Kruger’s strict criteria

We observed a significant increase in sperm morphological abnormalities following intravesical BCG induction therapy. After treatment, various defects in the head, neck, and tail were noted. We identified the amorphous head, short tail, and bent neck as the most frequently observed anomalies (Fig. 1).

## Discussion

Bladder cancer ranks among the most common urologic malignancies, with a notable increase in incidence correlating with advancing age. It occurs in men at a rate approximately four times higher than in women [1]. Bladder cancer predominantly affects older adults; however, younger patients, particularly those of reproductive age, encounter an added issue: the preservation of fertility. The effects of cancer therapies on male fertility are well-established, especially concerning chemotherapeutic agents recognized for their gonadotoxic properties. Nonetheless, the impact of intravesical BCG therapy on fertility is still inadequately investigated, even though there is evidence indicating a potential association between BCG-induced immune responses and reproductive dysfunction [8].

Numerous studies have examined the effects of BCG therapy on sperm quality, yielding inconsistent results. Animal models have produced inconsistent findings regarding the impact of BCG on testicular function. Naz et al. showed that a single intratesticular injection of BCG resulted in azoospermia within 3 to 6 weeks, with spermatogenesis gradually resuming after an extended duration of 153 to 325 days. Histopathological analysis showed aspermatogenic orchitis, leukocyte infiltration, and damage to the seminiferous tubules, while the basement membrane remained intact [9]. This indicates an immune-mediated mechanism influencing germ cell development. In contrast, Singh et al. catheterized the unilateral vas deferens lumen in an animal model and administered BCG, finding no significant effects on sperm morphology or motility [10]. The conflicting findings highlight the complexity of BCG's systemic effects and indicate a necessity for further investigation.

Research involving individuals has provided more direct evidence regarding the potential effects of BCG on male fertility. Raviv et al. conducted a study involving 12 men under 40 who were administered either intravesical mitomycin C (MMC) or BCG. The findings indicated that the MMC group exhibited no significant alterations in semen parameters, whereas 50% of the patients treated with BCG experienced a substantial decrease in sperm concentration (*p* = 0.0021). Furthermore, two patients demonstrated marked reductions in sperm motility, while one patient presented with significant morphological abnormalities [11]. Garg et al. conducted a prospective study involving 17 NMIBC patients aged under 45 years, revealing significant reductions in total sperm concentration (*p* = 0.0001) and motility (*p* = 0.0001). A total of 12 of the 17 patients exhibited a reduction in sperm concentration, with 5 cases of oligospermia and 7 cases showing decreased sperm motility. In contrast to our study, they did not report significant alterations in sperm morphology [12].

Our study revealed a statistically significant decrease in sperm concentration, total sperm count, and progressive motility, consistent with previous research findings. In accordance with earlier studies, our findings indicate a notable decline in normal sperm morphology along with an increase in morphological abnormalities (Fig. 1). These findings indicate that BCG-induced inflammation consistently influences sperm concentration and motility, whereas its effect on morphology appears to be more variable. The reasons for this discrepancy are not fully understood but may relate to variations in sample size, patient demographics, or individual immune responses.

### Potential mechanisms underlying BCG-ınduced reproductive changes

The precise mechanism through which intravesical BCG therapy affects sperm parameters is not completely understood. Several hypotheses have been proposed.

1. BCG-Induced Systemic Inflammation: BCG therapy is recognized for inducing local and systemic immune responses, characterized by the release of proinflammatory cytokines, including tumor necrosis factor-alpha (TNF-α), interleukin-6 (IL-6), and interferon-gamma (IFN-γ) [4]. Cytokines can induce oxidative stress and systemic inflammation, potentially extending beyond the bladder and negatively impacting testicular function. BCG-induced orchitis has been documented in case reports and animal models, reinforcing the hypothesis that immune activation influences spermatogenesis [12].
2. Disruption of the Blood-Testis Barrier (BTB): The BTB functions as a protective barrier, protecting developing germ cells from immune system-mediated damage. Systemic immune activation induced by BCG may compromise the integrity of the blood-testis barrier, resulting in heightened leukocyte infiltration in the testis and direct damage to seminiferous tubules, which subsequently impairs spermatogenesis [10].
3. Oxidative Stress and DNA Damage: Elevated inflammatory cytokines may contribute to increased oxidative stress in the testes, resulting in lipid peroxidation and fragmentation of sperm DNA. Research indicates a correlation between elevated oxidative stress and diminished sperm quality, alongside an increase in morphological abnormalities [8].

### Impact of age and additional variables

Our study found no statistically significant correlation between patient age and changes in sperm parameters. Some studies indicate that aging contributes to declining sperm quality due to oxidative stress and hormonal changes [2]. However, our findings suggest that the reproductive effects of BCG may be independent of age-related testicular decline. Larger studies encompassing diverse age groups are necessary to elucidate this relationship.

In addition to BCG therapy, various external factors may influence alterations in sperm parameters. Lifestyle factors including smoking, alcohol intake, dietary habits, and stress levels significantly influence semen quality. Our study excluded patients with diabetes mellitus, prior chemotherapy, prior genital surgeries, or radiation exposure to minimize confounding variables; however, we could not completely eliminate the potential impact of unmeasured factors, including subclinical inflammation or genetic predispositions. Future research must integrate detailed patient histories and biomarker analyses to address these variables.

### Limitations of the study

This study presents several limitations. The small sample size represents a significant limitation, potentially restricting the generalizability of the findings. Although a post-hoc power analysis confirmed that the sample size was adequate to detect significant changes in sperm parameters, a larger cohort would provide more robust statistical power and allow for subgroup analyses. Semen analysis was conducted once before BCG induction and once three months after treatment. The WHO advises conducting a minimum of two post-treatment semen analyses to verify substantial alterations in sperm parameters; therefore, depending on an individual post-treatment sample presents a limitation. Future research should incorporate multiple semen analyses at various time intervals to improve data reliability and evaluate potential variations in sperm parameters over time.

A significant limitation is the inability to measure free testosterone levels due to laboratory constraints. Total testosterone is often utilized as a marker of androgenic status; however, free testosterone provides a more precise representation of bioavailable androgen activity. The lack of assessment of free testosterone levels may have constrained our capacity to comprehensively evaluate the endocrine effects of BCG therapy. Future research should include free testosterone measurements to achieve a more accurate understanding of the hormonal changes linked to intravesical BCG treatment.

Additionally, this study focused on only the effects of the induction course of intravesical BCG, which includes six doses, without evaluating the potential cumulative effects of maintenance therapy. The impact of prolonged exposure to BCG in patients on long-term maintenance protocols on gonadotoxic effects remains uncertain. Future research should focus on comparing the reproductive effects of induction therapy and extended maintenance regimens to ascertain whether the observed changes in sperm parameters persist, decrease, or recover over time.

Furthermore, while we excluded patients with diabetes mellitus, prior chemotherapy, genital surgeries, or radiation exposure, we were unable to fully account for other confounding variables, including subclinical inflammation, oxidative stress, or genetic predispositions. A comprehensive assessment that includes inflammatory biomarkers such as C-reactive protein, IL-6, and TNF-α, along with oxidative stress markers, would yield valuable insights into the underlying pathophysiological mechanisms.

### Clinical implications and future directions

This study's findings highlight the potential effects of intravesical BCG therapy on male fertility, emphasizing the need for pre-treatment counseling and strategies for fertility preservation. Semen cryopreservation should be strongly considered for young NMIBC patients due to the significant reductions in sperm concentration, motility, and morphology. Patients must be made conscious of the potential gonadotoxic effects associated with BCG therapy and the significance of fertility preservation prior to the commencement of treatment.

Alternative intravesical therapies, including MMC and new immunomodulatory agents, may be appropriate for patients with important reproductive concerns. While MMC shows efficacy similar to BCG in intermediate-risk NMIBC, its effects on fertility are less thoroughly investigated. Future research should conduct direct comparisons of the reproductive effects of BCG and MMC, along with newer immunotherapies, to determine the most effective treatment strategy for NMIBC patients with fertility concerns.

Additionally, it is uncertain if BCG-induced changes in sperm parameters are reversible. Longitudinal studies with extended follow-up durations (e.g., 6 months, 12 months, and beyond) are essential to evaluate the recovery of sperm parameters over time following BCG therapy.

Future research should focus on the role of systemic inflammation and immune-mediated mechanisms in BCG-induced reproductive changes. Examining proinflammatory cytokine levels, oxidative stress markers, and testicular histopathology in NMIBC patients receiving BCG therapy could provide important insights into the behind pathophysiological mechanisms. Furthermore, research evaluating semen quality in NMIBC patients who cease BCG therapy following initial induction could explain whether discontinuing therapy allows sperm recovery.

Lastly, although our study focused on sperm quality, future research should also explore the potential impact of BCG therapy on other aspects of male reproductive health, such as erectile function, libido, and overall endocrine balance. Assessing gonadotropin-releasing hormone levels, adrenal androgen levels, and the hypothalamic-pituitary–gonadal axis may provide a more comprehensive understanding of how intravesical BCG affects male reproductive function.

## Conclusion

In summary, our research shows that intravesical BCG therapy has a significant impact on sperm concentration, motility, and morphology, suggesting possible gonadotoxic effects. These findings match with previous studies; however, our results indicate an important decrease in sperm morphology, a trend not extensively documented in earlier research. Based on these findings, it is essential to consider pre-treatment counseling, fertility preservation strategies, and long-term monitoring of reproductive function for NMIBC patients receiving BCG therapy.

Future research should aim to elucidate the mechanisms responsible for BCG-induced reproductive changes, evaluate the reversibility of sperm alterations, and investigate alternative intravesical therapies that could reduce gonadotoxic effects. Multicenter studies with longer follow-up periods are necessary to enhance clinical guidelines and improve fertility preservation strategies for NMIBC patients.

## Data Availability

The datasets generated during the current study are available from corresponding author on reasonable request. The data are not publicly available due to privacy or ethical restrictions.

## Abbreviations

NMIBC: Non-muscle-invasive bladder cancer
BCG: Bacillus Calmette-Guérin
TT: Total testosterone
FSH: Follicle-stimulating hormone
LH: Luteinizing hormone
PRL: Prolactin
MMC: Intravesical mitomycin C
BTB: Blood-testis barrier
